# Cell augmentation strategies for cardiac stem cell therapies

**DOI:** 10.1002/sctm.20-0489

**Published:** 2021-03-04

**Authors:** Raquel Cruz‐Samperio, Millie Jordan, Adam Perriman

**Affiliations:** ^1^ School of Cellular and Molecular Medicine, University of Bristol Bristol UK

**Keywords:** cardiac, cell migration, cellular therapy, clinical translation

## Abstract

Myocardial infarction (MI) has been the primary cause of death in developed countries, resulting in a major psychological and financial burden for society. Current treatments for acute MI are directed toward rapid restoration of perfusion to limit damage to the myocardium, rather than promoting tissue regeneration and subsequent contractile function recovery. Regenerative cell therapies (CTs), in particular those using multipotent stem cells (SCs), are in the spotlight for treatment post‐MI. Unfortunately, the efficacy of CTs is somewhat limited by their poor long‐term viability, homing, and engraftment to the myocardium. In response, a range of novel SC‐based technologies are in development to provide additional cellular modalities, bringing CTs a step closer to the clinic. In this review, the current landscape of emerging CTs and their augmentation strategies for the treatment post‐MI are discussed. In doing so, we highlight recent advances in cell membrane reengineering via genetic modifications, recombinant protein immobilization, and the utilization of soft biomimetic scaffold interfaces.

## Graphical abstract

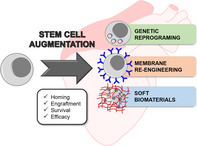

In this concise review, the landscape of emerging cell augmentation strategies for cardiovascular stem cell therapies are discussed. In particular, we highlight recent advances in cell membrane re‐engineering via genetic modifications, recombinant protein immobilization, and the utilization of soft biomimetic scaffold interfaces.

**Significance statement**

This review outlines the current challenges surrounding the adoption of stem cell therapies for the treatment of cardiovascular disease and the emerging technologies that are in preclinical development to overcome these hurdles. In doing so, the authors provide an overview of new approaches to stem cell membrane reengineering that aim to improve efficacy and reduce off‐target effects by improving homing and retention in the myocardium.

## 1 INTRODUCTION

Cardiovascular disease (CVD) accounts for 30% of fatalities globally and is the leading cause of mortality in middle‐to‐high‐income countries.^1^ In 2010, the global economic cost of CVD was USD 863 billon and it is expected to reach USD 1044 billion by 2030.^2^ One of the reasons for this disproportionate economic and societal burden is that the current treatments for myocardial infarction (MI), such as percutaneous coronary intervention (PCI) and coronary artery bypass surgery (CABG), are costly revascularization procedures which focus on managing the symptoms. PCI and CABG reduce the severity of the injury and the mortality; however, they fail to address myocardial structure and functional regeneration, which lead to costly follow‐ups, MI reoccurrence and death.^3^ The poor prognosis post‐MI can be attributed to limited self‐regenerative capacity of cardiac tissue after ischemic injury as cardiomyocytes (CMs) and cardiac stem cells (CSCs) die at infarcted site. CSC are then unable to undergo myogenic differentiation,^4^, ^5^ and scarring pathways are triggered instead to replace the dead CMs with viable myofibroblasts. This helps to maintain the myocardium structure by stimulating collagen I deposition, but fails to recapitulate the native tissue tensile strength and contractile forces that are required for functional left ventricle ejection pressure, prompting the reoccurrence of heart failure.^6^ For more information on the mechanisms and pathways associated with cardiac repair, the authors refer the reader to the comprehensive review by Broughton et al.^7^

Cell therapies (CTs) have emerged as promising regenerative treatments for range of different diseases, including CVDs (Table 1), but selecting the right cell type is essential for a successful outcome.^42^, ^43^ In early studies, myoblasts and skeletal muscle satellite cells were tested as potential CTs to treat MI, due to their capability to regenerate the muscle integrity of the heart,^8^ but clinical trials showed only minor improvements in ejection fraction performance and ventricular tachyarrhythmias.^12^ These cell phenotypes were superseded by bone marrow cells (BMCs),^13^, ^44^ especially bone marrow‐derived mesenchymal stromal cells (MSCs),^45^ which exhibit desirable properties for therapy, such as immunomodulatory capacity,^46^ a tendency to migrate to inflammation and injury sites, and multipotency.^47^, ^48^ Early preclinical studies suggested that MSCs could differentiate into a CM phenotype,^19^, ^20^, ^22^, ^49^, ^50^, ^51^ making them promising candidates for heart tissue regeneration. However, MSCs transplantation in animal models post‐MI,^19^, ^20^, ^22^ and in clinical trials, showed only modest levels of recovery of heart function.^12^, ^46^, ^52^ Indeed, later studies suggest that the observed therapeutic benefits result from paracrine effects^53^, ^54^ or acute immune responses,^21^ rather than MSC differentiation and subsequent engraftment. In response, recent preclinical studies have shown the regenerative potential of the secretome from different stem and progenitor cells (eg, MSCs, CSCs, embryonic cells, etc.) due to the cell‐specific complex mixture of cytokines, growths factors, enzymes, and genetic material,^55^, ^56^ leading to new technologies based on extracellular vesicles.^57^, ^58^ Although beyond the scope of the current review, the authors direct the reader to the comprehensive review by Xu et al^59^ for a detailed assessment of stem and progenitor cell secretomes, Levi et al^60^ for the scope and limitations of MSCs in CTs, and to Steinhoff^44^ for more information on other BMCs studied in cardiac repair.

**TABLE 1:** Cell therapies in cardiac therapies and their outcome in different animal models and clinical trials

Cells	Small mammal models	Swine models	Primate models	Clinical trials
Myoblasts	Aut.,^a^ muscle regeneration^8^ Aut., enhanced oxygenation, contractile function recovery^9^	Al.,^b^ cell survival for 10 days.^10^ Al., paracrine effects on ECM remodeling and vascularization.^11^	‐	Aut., ventricular tachyarrhythmias^12^
BMCs^c^	Al., improved tissue regeneration^13^ Aut., enhanced angiogenesis after a week^14^	Aut., improved cardiac function, and higher blood flow and capillary function after 3 wk^15^	Aut., improved regional blood flow and cardiac function via paracrine effects^16^	Aut., improved infarct tissue perfusion and left ventricular function^17^ Aut., decreased infarct size, improved left ventricular function^18^
MSCs^d^	Al., heart regeneration via differentiation into CMs^19^ Al., myocardium repair via paracrine effects^20^ Aut., improved ventricular performance via acute immune response^21^	Aut., structural and functional remodeling^22^ Al., angiogenesis, reduction of infarct size, improved contractile function via trilineage cell differentiation^23^	‐	Aut. and al., enhanced ventricular remodeling and functional capacity^24^
MSCs‐CSCs^e^	Aut., decreased infarct size, improved cardiac function via paracrine effects^25^	Aut., scar size reduction^26^ Al., scar size reduction and systolic function recovery^27^	‐	Aut., undergoing^28^
iPSCs‐CMs^f^	Aut., improved left ventricular function^29^ Al., improved left ventricular function^30^	Al., improved contractile function^31^	Al., contractile function improvement^32^	Al.^33^ and Aut.,^34^ transplanted in cell sheets, undergoing
hESC‐CMs^g^	Al., CMs survived and engrafted to the heart for weeks^35^, ^36^ Al., angiogenesis and ECM^h^ formation^37^	Al., adequate engraftment^38^	Al., enhanced remuscularization^39^ Al., improvement of left ventricular function^40^	Al, transplanted in fibrin patch, improved systolic function ^41^

Abbreviations: BMC, bone marrow cell; CMs, cardiomyocytes; CT, cell therapy; ECM, extracellular matrix; hESC, human embryonic stem cell; iPSCs, induced pluripotent cells; MSC, mesenchymal stromal cell.

^a^ Autologous CT (cell source is the patient).

^b^ Allogenic CT (cell source is different than the patient).

^c^ Bone marrow cells.

^d^ Mesenchymal stromal cells.

^e^ Mesenchymal stromal cells combined with cardiac stem cells.

^f^ Induced pluripotent stem cells differentiated into cardiomyocytes.

^g^ Human embryonic stem cells differentiated into cardiomyocytes.

^h^ Extracellular matrix.

MSCs are by no means the only SCs in the spotlight for cardiac tissue regeneration, as promising preclinical results have also been achieved using induced pluripotent cells (iPSCs)^61^ and human embryonic stem cell‐derived CMs (hESC‐CMs).^39^, ^40^, ^62^ iPSCs have been shown to be plausible candidates for cardiac therapy in several animal models.^61^, ^63^, ^64^ Transplantation of iPSCs predifferentiated into CMs has been reported to ameliorate ventricular function in rodent models,^29^, ^30^ and contractile function in porcine models.^31^ iPSC‐CMs have also been developed as a potential allogenic CT and tested in nonhuman primates, displaying electrical integration with the host heart, leading to improvement of contractile function after 4 weeks with no significant immune rejection.^32^ These encouraging preclinical results supported clinical trials to determine the safety to iPSC‐CM transplantation as cell sheets.^33^, ^34^

In vivo CT studies to treat cardiac ischemic injury from hypoxia have postulated a wide range of possible regenerative mechanisms that are linked to cell type and origin (autologous or allogenic). These include functional integration with recipient CMs, activating the growth and differentiation of endogenous CSCs,^65^, ^66^ *trans*‐differentiation of transplanted SCs into new CMs and/or endothelial cells, metalloprotease‐driven cardiac tissue matrix remodeling, as well as via the recruitment of white blood cells to repair micro‐vessels.^67^, ^68^ Unfortunately, when transplanted via intravenous or intra‐arterial infusion, SCs accumulate in tissue sinks such as the lungs and liver, which reduces the efficiency of systemic delivery and increases the likelihood of producing lethal microemboli.^69^, ^70^, ^71^, ^72^, ^73^ Even when implanted directly into the region of interest, the number of cells required for therapeutic benefit may be prohibitively high. Indeed, cell tracking experiments performed on a range of cells injected into the infarcted hearts of mice displaying cell necrosis have shown limited long‐term integration, with only 2% to 10% of cells remaining over the first few days and virtually none after 3 months.^74^, ^75^ Such low levels of engraftment and retention can be rationalized by a number of factors, including the lack of cell adhesion, turbulent flow, hypoxic microenvironments, and the presence of inflammatory cytokines.^76^, ^77^, ^78^

It is becoming evident that irrespective of their source, therapeutic stems cell need to be recruited/retained near the injury site in significant numbers and duration to have a positive clinical outcome.^47^, ^79^, ^80^ Accordingly, augmenting SCs with soft biocompatible interfaces that drive homing and engraftment to cardiac tissue could rapidly accelerate the rate at clinical translation. This review explores the current and emerging methodologies in cell augmentation technology for enhancing the performance of cardiac CTs.

## 2 APPROACHES FOR IMPROVING SC HOMING AND RETENTION

**FIGURE 1:**
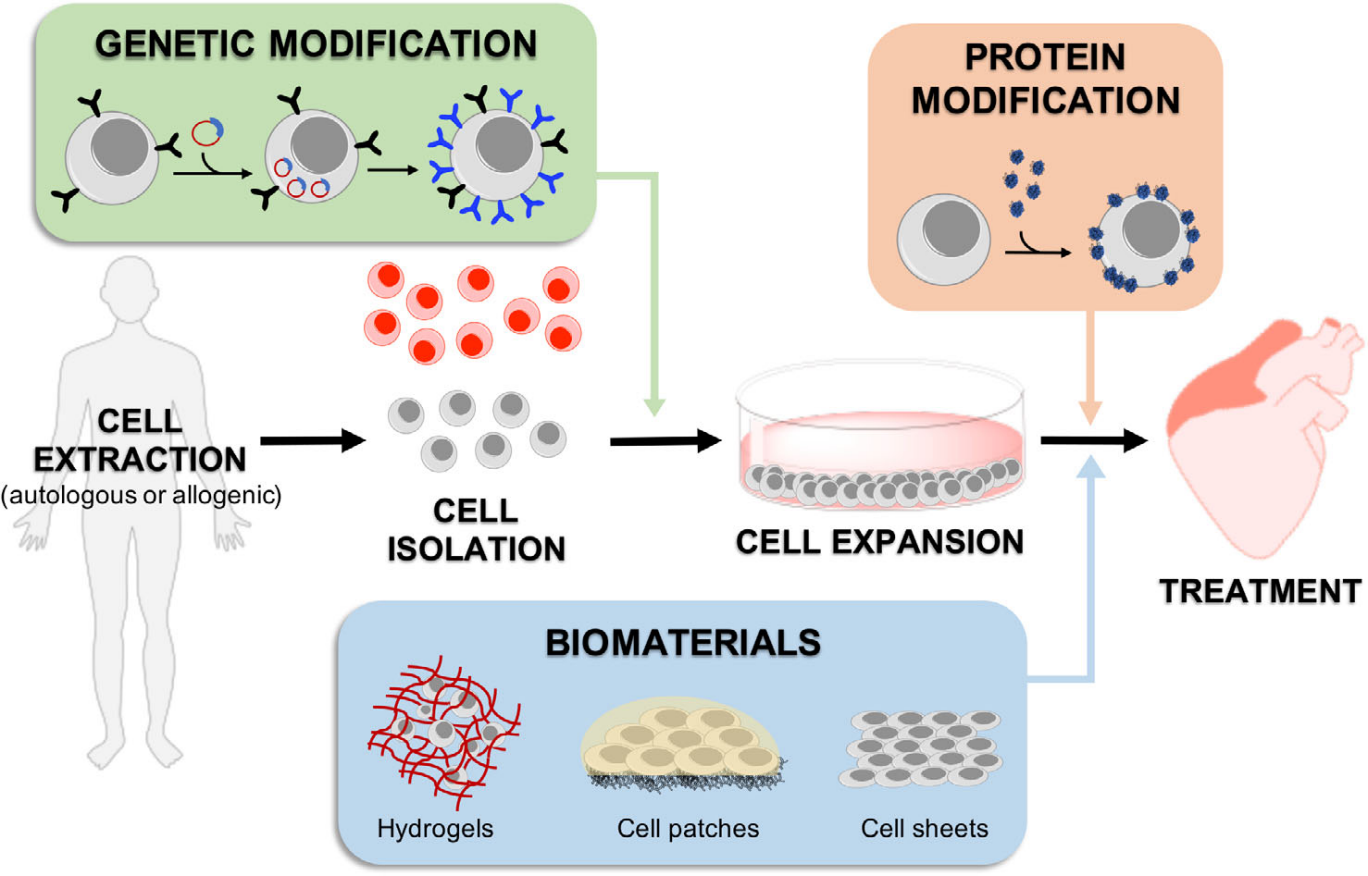
Methods for augmenting stem cell (SC) to improve homing and retention in cardiac therapies. SCs are extracted from the source, purified, and expanded to achieve the desired numbers for treatment. Their cell membrane can be modified to improve homing to cardiac tissue genetically, or by using homing proteins or soft biomaterials. SCs can be genetically modified prior to the expansion phase to overexpress membrane receptors or adhesion markers. They can also be treated with proteins that are prone to stick to cardiac tissue after expansion or they can be built into scaffolds that provide them with new functionalities and properties

In general, cell homing and adhesion to cardiac tissue can occur via specific receptor‐mediated interactions with the extracellular matrix (ECM) proteins, or can be initiated via physical/chemical adhesion to cardiac cells via hydrophobic and electrostatic interactions. It is widely accepted that SCs target inflammation and injury sites, including infarcted myocardium. Accordingly, many SC membrane reengineering approaches aim to promote these endogenous processes, but there is a growing body of literature that describes the development of new targeting strategies to improve homing efficiency (Figure 1).

### 2.1 SDF‐1/CXCR4 axis

SCs, such as MSCs, iPSCs, or ESCs, possess a set of cell adhesion markers (eg, integrins ß1, ß2 [CD18], or VL‐4), growth factors (eg, glycoprotein CD34^+^ or vascular endothelial growth factor [VEGF]) and chemokine receptors, which play an important role in cell homing to sites of inflammation.^80^ In particular, MSCs can come naturally to the bone marrow niche through binding of the extracellular C‐X‐C chemokine receptor type 4 (CXCR4) to the stromal cell‐derived factor 1 (SDF‐1), which is present in the bone marrow.^81^, ^82^ This interaction could be readily exploited for cardiac therapies, as SDF‐1 expression is upregulated at the injury site over 48 hours post‐MI,^80^ but unfortunately the MSC expression of CXCR4 is commonly downregulated or lost during their expansion.^83^ Several approaches have been reported to upregulate CXCR4 expression in MSCs during expansion, such as culturing under hypoxic conditions.^84^ Moreover, treating MSCs with growth factors^81^ such as insulin‐like growth factor 1 (IGF‐1),^85^ tumor necrosis factor α,^86^ interleukin 1ß,^87^ interferon γ,^88^ or pretreating with chemokines (glycogen synthase kinase 3ß),^89^ can increase CXCR4 expression. Despite these efforts, CXCR4 expression levels are generally not sufficient to promote high levels of homing, and endogenous proteases, such as matrix DPP‐4/CD26, degrade this receptor, promoting the loss of MSCs in the myocardium.^90^

### 2.2 Genetically modified SCs

Improving the therapeutic potential of a SC can be achieved genetically using viral or liposome‐based vectors,^91^ resulting in overexpression of the protein of interest (Figure 1). Genetic modification of SCs have focused mainly on increasing paracrine factor production, inducing differentiation into CMs or improving retention or integration with the heart in cardiovascular diseases.^91^, ^92^ Chen et al and Zhang et al parallelly showed that retrovirus‐ or adenovirus‐induced overexpression of CXCR4 in MSCs resulted in a respective decrease in anterior wall thinning and left ventricular remodeling,^93^ and an increase in angiogenesis and myogenesis^94^ when transplanted in rats post‐MI. Similarly, overexpression of SDF‐1, the CXCR4 natural counterpart, in transplanted MSCs has shown increased recruitment of endogenous SCs, leading to a 20% decrease in fibrotic area and 20% increase in ejection fraction compared to saline in rats.^95^ However, rather than targeting homing, most studies on MSC genetic modification have focused on improving their therapeutic potential by either augmenting their paracrine factor production^96^, ^97^, ^98^, ^99^, ^100^, ^101^ or by facilitating their differentiation into CMs.^102^

Some of the first examples in the literature displayed enhanced MSC survival after implantation in ischemic rat myocardium by retrovirally inducing overexpression of pro‐survival factors, such as Akt^96^ or Bcl‐2,^97^ leading to reduced inflammation and up to 90% recovery of myocardial volume and cardiac performance for the former (Akt) and 32% increased survival of the implanted MSCs, causing a 17% reduction of the infarct size for the latter (Bcl‐2). Other approaches induced overexpression of angiogenesis factors in MSCs, such as VEGF^98^, ^99^ or GATA‐4.^100^ VEGF‐expressing MSCs have been reported to promote angiogenesis and improve the infarct size after a month by 10% in rats^98^ and 30% in sheep^99^ post‐MI, whilst GATA‐4 triggered antiapoptotic pathways when overexpressed in MSCs, displaying a threefold increase in ejection fraction and fractional shortening compared to unmodified MSCs in mice after MI.^100^ Directly overexpressing the growth and transcription factor regulator, thioredoxin‐1, in MSCs improved proliferation by 20%, and most importantly, the production of VEGF, heme oxygenase‐1 (HO‐1) and CXCR4 after 4 days from implantation in rats, resulting in improved contractility and ejection fraction after 60 days.^101^ Finally, MSC differentiation into CMs has been achieved via overexpressing the cardiomyogenic transcription factor myocardin before transplanting the cells in mice post‐MI. MSCs overexpressing myocardin displayed enhanced engraftment with the heart and recovered left ventricular function after 15 days from treatment.^102^

Overall, genetic modification is a versatile and exciting approach to enhance MSCs therapeutic performance and retention in the heart; however, the cost associated with reprogramming can be prohibitively high. Moreover, the modifications are permanent and the use of viral vectors is subject to insertional oncogenesis.^47^

### 2.3 Protein‐based membrane modifications

Direct protein‐based membrane modification strategies provide transient display of the targeting construct and present a number of potential benefits over generic approaches for improved cell homing. These include, a reduction in risk arising from oncogenesis in the therapeutic cells, minimal impact on the cell manufacturing process as the modification step can be readily integrated into an existing therapeutic pipeline, the display number per cell can be systematically varied to reduce the risks associated with patient‐specific expression levels, and protein production is scalable and can be produced using good manufacturing practice procedures.^103^, ^104^

**FIGURE 2:**
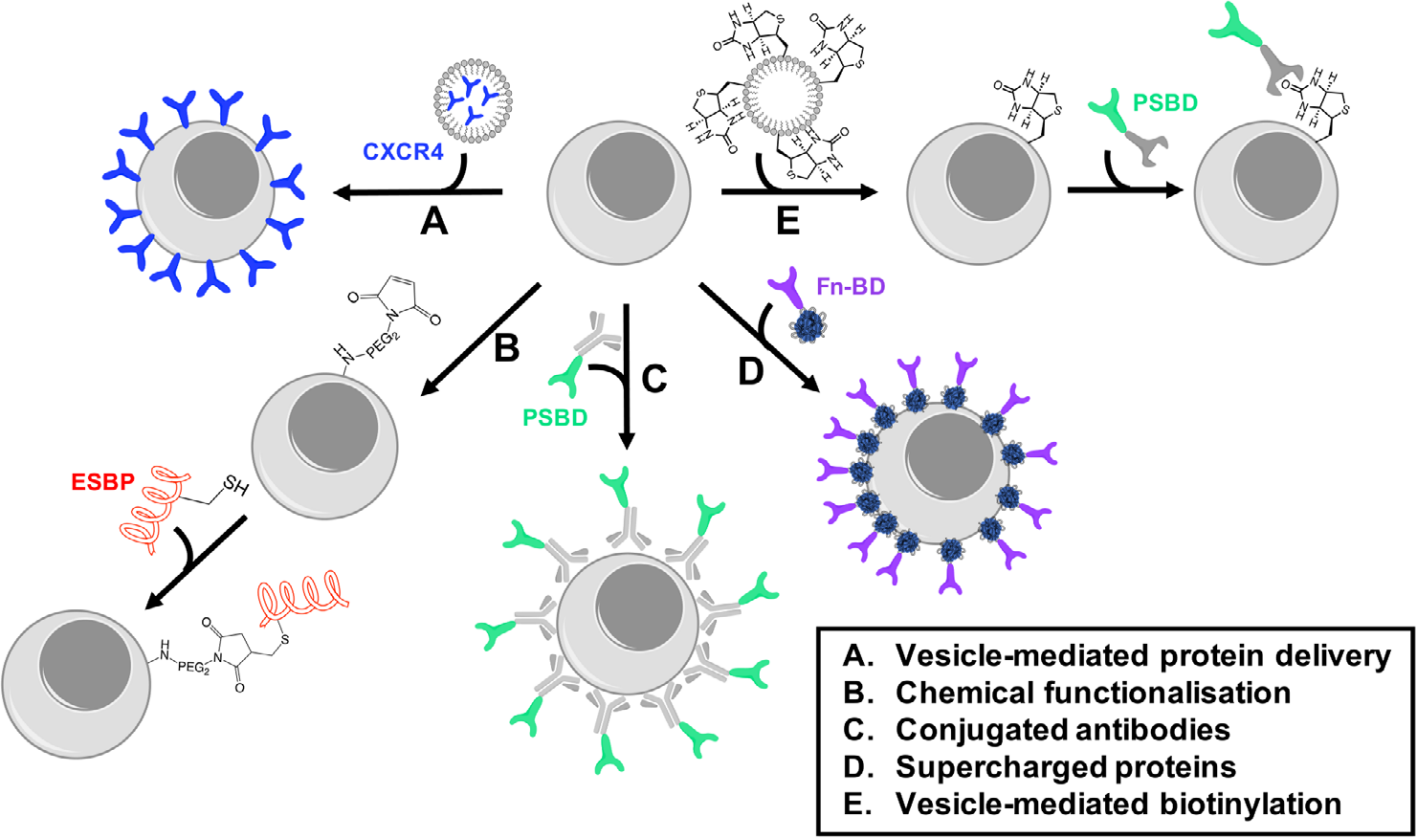
Protein‐based reengineering stem cell (SC) membranes to improve homing in cardiac therapies. A, Vesicle‐mediated CXCR4 delivery to insert ligand in the membrane of SCs. B, Chemical functionalization of free amines to covalently attach E‐selectin binding peptide (ESBP) to SC membrane. C, Conjugating antibodies to P‐selectin binding domain (PSBD) to deliver ligand to membrane through antibody‐epitope interactions. D, Surfactant‐coated supercharged proteins conjugated to fibronectin binding domain (Fn‐BD) strongly interact with SC membrane. E, Vesicles decorated with biotin merge with the cell membrane of SCs to allow further functionalization of SCs with proteins, such as PSBD, conjugated to streptavidin

An excellent example of direct protein‐based membrane modification was demonstrated by Won et al, who displayed recombinantly produced CXCR4 on MSC membranes using lipid‐PEG vesicles (Figure 2A).^105^ They demonstrated that CXCR4 was only present in the MSCs membrane after delivery by confocal microscopy studies without affecting the viability of MSCs and showed up to a twofold improvement in their migration toward SDF‐1 following a concentration gradient in vitro. This noninvasive approach allowed facile reengineering of MSC membranes within 2 minutes, which is especially relevant for autologous therapies, considering that genetic modification methods can take several weeks, missing the ideal therapeutic window for treating the infarcted myocardium.

Besides the SDF‐1/CXCR4 axis, homing to ECM adhesion proteins overexpressed in inflammation sites, such as selectin and fibronectin, has also been investigated.^106^, ^107^, ^108^ There are several avenues to achieve this, one being the covalent conjugation of MSCs to E‐selectin binding peptides in a two‐step process (Figure 2B). Here, the free amine groups in MSC membrane proteins are functionalized with NHS‐PEG_2_‐maleimide, and then ligated to free thiols of the E‐selectin binding peptide. This resulted in successful SC adhesion and rolling on immobilized E‐selecting under up to 0.5 dyn/cm^2^ sheer stress in vitro, without affecting cell viability, proliferation, or multipotency.^106^ However, direct covalent modifications of free amines on cell surfaces lacks target membrane protein specificity and could give rise to downstream toxicity or immunogenicity. Moreover, plasma membrane proteins are involved in many different signaling cascades, and their modification could potentially affect MSCs fate and subsequent therapeutic performance. Accordingly, other approaches aim to only modify the plasma membrane non‐covalently. One approach involves decorating a human antibody (IgG1) with P‐selectin glycoprotein ligand‐1 (Figure 2C). The modified IgG1 binds to the MSCs cell membrane, conferring the MSCs enhanced adhesion and rolling to P‐selectin and E‐selectin 1 and 2 dyn/cm^2^ shear stress, respectively. The therapeutic applicability of this method was demonstrated by showing MSC retention to human umbilical vein endothelial cells (HUVECs) under static (up to 10 dyn/cm^2^) and hydrodynamic shear (up to 4 dyn/cm^2^).^107^ Similarly, a recent study by Wu et al demonstrated that antibodies could be used to direct MSCs to cardiac tissue via membrane biotinylation and binding to a streptavidin‐conjugated antibody specific to inflamed endothelium.^109^ This approach conferred MSCs with a twofold increase in retention to an ischemic myocardium without diminishing MSC cell viability in a mouse model. Moreover, the authors also demonstrated target specificity by subjecting antibody‐modified and nonmodified MSCs to HUVECs coated with the antibody epitope under physiologically relevant wall stress shear, obtaining the same trends observed in the mouse model.

Recently, Perriman et al developed a noncovalent methodology to rapidly display proteins and enzymes on the plasma membrane of mesenchymal SCs.^108^, ^110^, ^111^ These designer proteins comprise supercationic protein‐polymer surfactant plasma membrane binding domains that spontaneously assemble at the cell surface (Figure 2D). When the team applied the methodology in the field of targeted cardiac CTs, they demonstrated that the inherent cardiac homing properties of the oral bacterial *Streptococcus gordonii* could be transferred to hMSCs through the rational design of a membrane active bacterial adhesin protein chimera. Here, the fibronectin binding domain of the bacterial adhesin CshA was expressed as a fusion with supercharged green fluorescent protein and the resulting modified hMSCs showed a twofold increase in number of cells in myocardium after either intravenous or intracardiac injection in a murine model, without a commensurate increase in the lungs.

Another excellent example of direct plasma membrane modification involves the application of biotinylated lipid vesicles, which has been used to coat MSCs with biotin for subsequent functionalization with biotin‐binding moieties (Figure 2E). Here, biotin's high binding affinity for streptavidin was exploited to attach streptavidin‐conjugated P‐selectin homing ligands to MSCs, enhancing MSC rolling interactions to P‐selectin under up to 0.75 dyn/cm^2^ dynamic flow conditions.^103^ The best P‐selectin interaction was obtained at 0.5 dyn/cm^2^ shear stress, where 80% of modified MSCs showed interaction vs only 32% nonmodified MSCs, but this difference became rapidly smaller at higher forces. Reengineering MSCs membrane with biotinylated lipid vesicles is an appealing, versatile method to modify cell membranes as any protein conjugated to streptavidin can be implemented and the authors demonstrated that the modified vesicles had no negative impact on MSC viability, adhesion to polystyrene surfaces or MSC multipotency.

The nongenetic approaches to cell membrane modification discussed above highlight the breadth of approaches for reengineering SCs membranes, and for a more in‐depth look at the field, the authors direct the reader to the comprehensive reviews by Lee et al^112^ and Armstrong et al.^113^ What is becoming clear, however, is that despite the fact that the approaches lead to an increase in target affinity, there is still a lack of compelling preclinical data to support clinical translation. Even so, cell membrane reengineering using proteins is an exciting methodology to augment SCs and next‐generation new protein ligation tools are emerging,^114^, ^115^ which will allow rapid bioorthogonal functionalization of SCs to instill a range of new properties and cellular functions.

## 3 SOFT BIOMATERIALS FOR SC DELIVERY

**FIGURE 3:**
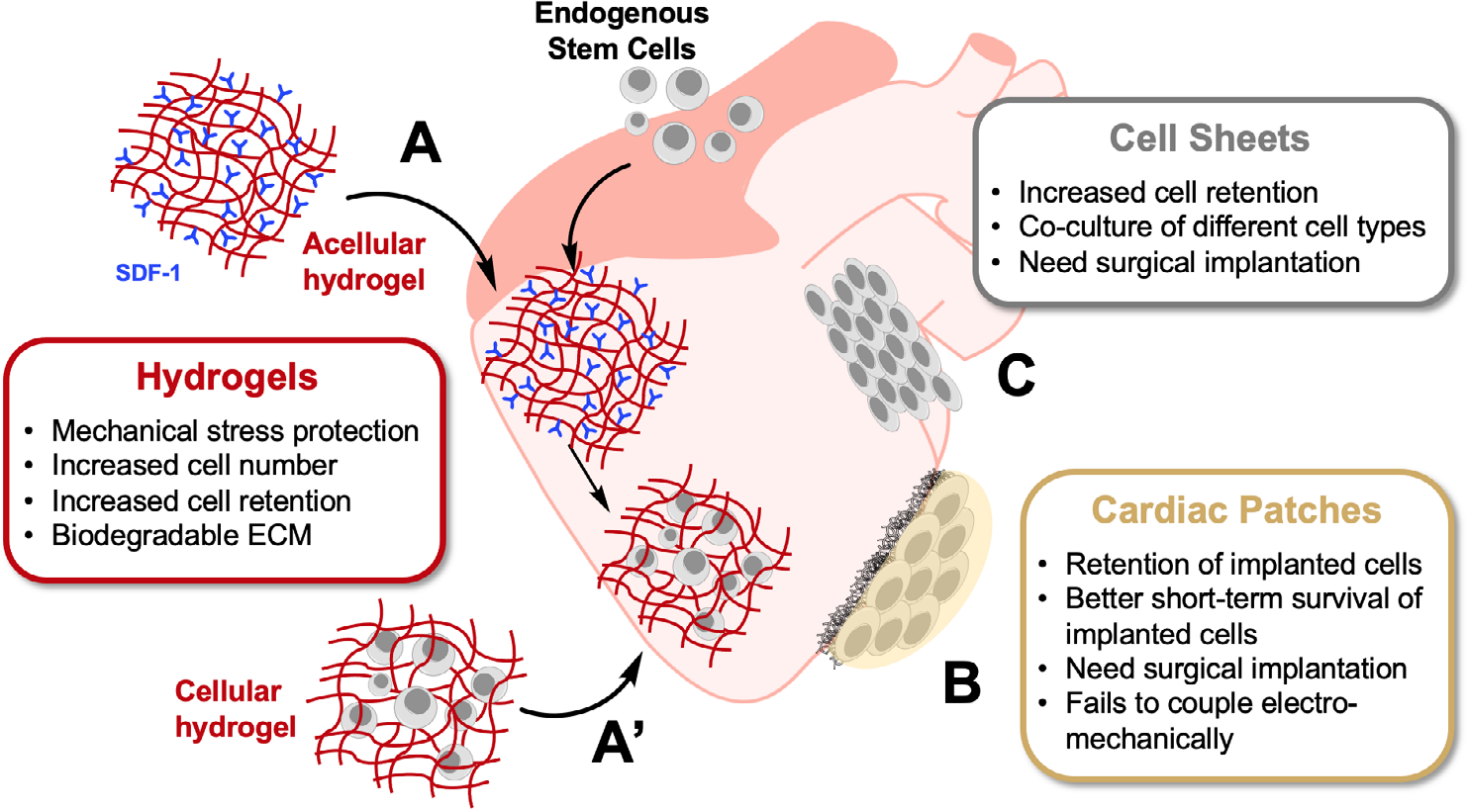
Biomaterials can provide stem cells (SCs) with an ECM‐like microenvironment to promote adhesion and retention in the myocardium and, ultimately, to enhance SC therapeutic outcome. A, Acellular hydrogels modified with specific factors (eg, SDF‐1) recruit endogenous SCs after transplantation. A', Cellular hydrogels protect the implanted cells from mechanical stress from the injection. B, Cardiac patches offer the best short‐term protection and retention, but they are more rigid than the other options and usually fail to couple electromechanically with the heart. C, Cell sheets can contain monolayers of single cell types or coculture of different types to contribute to different processes involved in cardiac repair

Despite not being strictly a direct cell membrane modification approach, soft biomaterials can provide an ECM‐like environment to the transplanted SCss, which can have a major impact on cell adhesion at an infarcted site, cell survival and retention in the myocardium, and hence the overall efficiency of the treatment. Accordingly, the application of injectable hydrogels, cell patches and cell sheets, and their respective performances are discussed below (Figure 3).

### 3.1 Hydrogels as transplant matrices

The incorporation of injectable hydrogels in CTs has drawn much attention in the last decade, as they provide a biocompatible three‐dimensional matrix to the transplanted cells (Figure 3A) and form the basis for the majority of bioinks used in 3D bioprinting.^116^, ^117^ Preclinical cardiac CT studies have utilized a range of biologically derived hydrogels from mammalian sources, such as collagen, and polysaccharides like hyaluronic acid (HA), as well as other nature‐derived examples, which include chitosan (from seafood industry waste) or alginate (from seaweed), which are biodegradable and have similar mechanical properties to the infarcted tissue.^118^ Several key examples have shown that acellular injectable hydrogels of different compositions are safe for cardiac implantation in murine models.^119^, ^120^, ^121^ These studies also showed an increase in endogenous BMCs homing to the heart after injection of HA hydrogels modified with recombinantly expressed SDF‐1^119^, ^120^ or with collagen I hydrogels embedded with histone deacetylase 7 peptide.^121^ In both cases, enhanced angiogenesis and recovery of left ventricular function were observed, and infarct size decreased up to fourfold in comparison with the control models. Recent clinical trials have reported that injecting patients suffering from heart failure with acellular alginate hydrogels is safe and increased the rate of recovery when combined with standard treatments.^122^

The scope of using hydrogels in cardiac therapies is not limited to acellular transplantations, as many efforts report successful delivery of embedded cells in hydrogels to infarcted tissue in animal models (Figure 3A')^123^, ^124^, ^125^, ^126^ and clinical trials.^127^ Hydrogels not only can be transplanted as matrices, but can also be directly injected and have been shown to protect the transplanted cells from the mechanical shear of injection^128^ and increase the cell number and retention at the targeted tissue.^129^ Early clinical trials in patients with ischemic injury involved injecting autologous BMCs embedded in a collagen I hydrogel, however, despite proving to be safe for the patients, no major improvement in heart function was observed.^127^ New efforts have focused on improving the efficacy of these CTs by the addition of signaling ligands (eg, Notch ligand delta‐1)^123^ and peptides derived from growth factors (eg, insulin‐like growth factor 1, angiopoietin‐1)^124^, ^125^ to enhance the survival of the transplanted cells, and by improving the mechanical properties of the gel.^126^, ^130^ A recent study has reported successful implementation of hESC‐CMs in a collagen hydrogel modified with recombinant Notch ligand delta‐1 in rats.^123^ The Notch signaling ligand doubled both the proliferation rate of the implanted hESC‐CMs and the graft size compared to the controls, even when the cells were transplanted in subtherapeutic numbers.

Another key study has shown that embedding the C‐terminal domain peptide of IGF‐1 in chitosan hydrogels containing MSCs improved cell survival by threefold in mice by protecting the transplanted cells from oxidative stress, resulting in enhanced angiogenesis by over 60%, 30% reduction in collagen deposition, and general improvement of cardiac function.^124^ Improving the hydrogel delivery to reduce mechanical stress during injection could also have a beneficial effect on the survival of the transplanted cells. Endothelial progenitor cells (EPCs) have been reported to enhance vasculogenesis by a fourfold in rats when implanted in HA hydrogels, which exhibit enhanced shear‐thinning properties and hence improved delivery via injection.^126^, ^130^ A deeper understanding on how to rationally design hydrogels to improve the efficacy of transplanted CTs is still required before they can be effectively translated to the clinic, but advances in this field are to be expected after the initial clinical trials have endorsed their safety. One of the main limitations of these approaches remains the immune rejection of the transplanted graft. Nevertheless, a recent exciting report of Kim et al. describes how this may be overcome by encapsulating regulatory T‐cells (Tregs) cocultured with murine pancreatic islets in alginate‐gelatin methacryloyl hydrogels for the treatment of type I diabetes mellitus.^131^ This approach should be explored in cardiac repair grafts when considering that Tregs have been shown to be safe in clinical trials and are currently supplemented during liver transplants to suppress immune rejection.^131^ For more information on Tregs and their mechanism of suppression graft immune rejection, authors refer the reader to the review by Romano et al.^133^

### 3.2 Cardiac patches

Cardiac patches have emerged as a potential solution for the poor retention and survival of transplanted cells in the heart (Figure 3B). Here, SCs or SC‐derived CMs, are grown in vitro and then adhered to a scaffold that suits the size of the injury, and that has a matrix that allows oxygen diffusion and resistance to contractile forces. Once the desired cell confluency is achieved on the patch, it is surgically implanted. The main limitation of this exciting approach is that is it difficult to integrate electromechanically and immunologically within the heart and the transplanted cells display low long‐term survival in animal models.^134^ For example, initial phase I clinical trials have reported short‐ and medium‐term safety of transplanting hESCs in fibrin‐based patches in six patients, but no information on the hESC survival rate or the patch electromechanical coupling was recorded.^41^ Phase I clinical trials to determine the safety of hESC collagen patches have been completed in November 2020, but the outcome is yet to be published.

In an effort to overcome some of the limitations of cardiac patches, novel next‐generation designs are emerging, which include cellulose nanofibers MSC patches to enhance neovascularization of infarcted myocardium in rats,^135^ porous polymeric polyvinyl alcohol microneedles to ameliorate nutrient flow between the CSC patch and the myocardium in rats and swine post‐MI,^136^ and overexpression of cell proliferation factor, hepatocyte growth factor, in the transplanted MSCs to maintain constant cell numbers in the cardiac patch despite the hypoxic environment in porcine post‐MI models.^137^ Moreover, efforts are being made to reduce the size of the constructs to alleviate the need for invasive surgeries, for example, the development micro‐scale hESC‐CMs patches via intramyocardial injection in rats, which also improved their electromechanical coupling to the host heart.^138^

Another limitation of this technology is that cardiac patches need to be freshly prepared to ensure cell viability and functionality of the transplanted cells, and thus, they are unavoidably transplanted few days after the infarction event, compromising the efficacy of the treatment. A recent paper by Huang et al., presented the first example of a promising acellular, off‐the shelf cardiac patch that can be easily prepared by mixing porcine decellularized myocardial ECM and a solution of synthetic CSCs factors embedded in biodegradable microparticles.^139^ This patch reduced scarring and promoted angiomyogenesis by 40% and left ventricular ejection fraction by 15% in rats and was shown to be safe in pigs, even after been cryopreserved for 4 weeks prior to the study. Further studies are needed to determine its safety and efficacy in patients, but it is a promising avenue for cardiac patch development.

### 3.3 Cell sheet technologies

Cell sheet technology is an attractive alternative to cardiac patches, as the resulting structures exhibit high cell concentration and uniformity, confer more resistance to degradation upon implantation, and only rely on the formation of tight cell‐to‐cell junctions and ECM protein secretion, rather than an artificial scaffold (Figure 3C).^140^ Cell sheets are prepared by culturing monolayers of cells on temperature‐responsive substrates, which become nonadherent at low temperatures,^141^ and provides the opportunity to produce cellular multilayers through direct manipulation (up to three layers) or sequential assembly on a hydrogel‐coated plunger (up to five layers).^142^ Once the cell sheet is formed, it detaches from the substrate via a temperature change, which allows for efficient and effective surgical implementation. The approach has been used to transplant a wide range of cells to infarcted myocardium, including autologous myoblasts,^143^ autologous skeletal cells,^144^ allogenic cardiac progenitor cells^145^ and allogenic iPSC‐CM.^146^ Overall, the approach has been shown to enhance cardiac regeneration in several animal models when compared with the direct injection of cell suspensions, possibly due to retention of higher cell numbers on the heart and the formation of tight cellular junctions within the sheets. There are currently allogenic iPSC‐CM cell sheets undergoing clinical trials to determine their safety and efficacy on patients with chronic ischemic cardiopathy combined with bypass graft surgery.^33^

Cell sheet technology also provides a clear pathway for the development of cellular structures with multiple cell types, which can contribute collectively during cardiac regeneration. For example, fibroblasts are essential for the maintenance of the ECM environment, endothelial cells for the formation of new capillaries, smooth muscle cells for neovascularization and neurons for autonomic control to the heart.^147^ Early examples are the transplantation of fibroblast sheets cocultured with EPCs^148^ and EPC sheets cocultured with CMs^149^ in rats post‐MI, respectively. Both studies reported a 10‐ and 2‐fold increase in vessel formation respectively, linked to the presence of EPCs in the graft, compared to the monoculture counterparts, but also reduced the formation of fibrotic tissue when compared to EPC grafts, suggesting that the fibroblasts and CM key players in the tissue regeneration. Similarly to EPC cocultures, a recent example has combined iPSC‐CM sheets with the vascular‐rich pedicle omentum flap to enhance the endurance of the graft through improvements the blood supply in a mini‐pig MI model.^146^ iPSC‐CM cell sheets improved the cardiac function after a month in the presence and absence of the omentum; however, the graft combined with the omentum was reported to augment the capillary density by twofold, upregulate paracrine factors (eg, VEGF, SDF‐1) and promote CM maturation after 3 months, compared to the iPSC‐CM sheet implantation only. More studies to determine the safety and therapeutic potential of cell sheets are needed, but cocultures of different cell types seem to be the most promising avenue.

## 4 CONCLUSION AND FUTURE PERSPECTIVES

CTs are now starting to emerge as a credible alternative to current MI treatments as they address cardiac repair via activation of endogenous SCs (CSCs, MSCs, etc.) or via engraftment into the heart. However, many of the challenges that reduce their efficacy still remain, such as poor long‐term cell survival, limited homing, tumor formation, and lack of retention in the infarcted myocardium. Nevertheless, the fast‐growing development of new technologies to reengineer the membrane of SCs or provide a supporting biocompatible matrix may alleviate these limitations. It is clear that genetic approaches are extremely exciting, as they can be implemented through reliable protocols that are easy to track in the preclinical phase (eg, co‐expression of fluorescent proteins), and have a low risk of triggering unwanted immune responses. However, the risk of mutation‐derived oncogenesis is still a concern, which paves the way for transient non‐genetic SC modification approaches. Another potential limitation is that current reprogramming methodologies are not temporally compatible with autologous CTs, as the expansion phase is lengthened by several weeks, missing the ideal therapeutic window after MI. Similarly, cardiac patches and cell sheets also require extended culture periods. This cell number challenge could be overcome with the development of an allogenic CT, giving rise to a readily accessible off‐the‐shelf treatment, which could be subjected to high quality control processes.^24^, ^150^

With respect to the developments within the biomaterial scaffold space, although they offer an effective solution to myocardium cell retention and cell number, the transplant process is generally more invasive, and challenges with effective electromechanical integration still remain. Shear thinning injectable hydrogels have great potential, as they are generally less invasive, offer protection to the transplanted cells from mechanical stress, and provide a rudimentary micro‐ECM that can be systematically tuned for cell signaling. It is also worth highlighting efforts on transplantation of acellular hydrogels and cardiac patches that attract endogenous stem/progenitor cells and provide them with a scaffold to promote long‐term survival. Moreover, these scaffolds could be modified or implemented in combination with molecules that activate the recruited cells (eg, statins or TGF‐β/Wnt signaling molecules) and amplify their therapeutic potential. In conclusion, it is likely that no single approach to SC membrane reengineering will provide the “magic bullet” for cardiac CTs, and that the next generation of therapies will likely utilize combinations of these technologies to fully harness the therapeutic potential of transplanted SCs.

## CONFLICT OF INTEREST

The authors declared no potential conflicts of interest.

## AUTHOR CONTRIBUTIONS

R.C.S.: conception and design, manuscript writing; M.J.: manuscript writing; A.P.: conception and design, manuscript writing, final approval of manuscript.

## Data Availability

Data sharing is not applicable to this article as no new data were created or analyzed in this study.
